# Transcriptome Exploration in *Leymus chinensis* under Saline-Alkaline Treatment Using 454 Pyrosequencing

**DOI:** 10.1371/journal.pone.0053632

**Published:** 2013-01-24

**Authors:** Yepeng Sun, Fawei Wang, Nan Wang, Yuanyuan Dong, Qi Liu, Lei Zhao, Huan Chen, Weican Liu, Hailong Yin, Xiaomei Zhang, Yanxi Yuan, Haiyan Li

**Affiliations:** 1 Ministry of Education Engineering Research Center of Bioreactor and Pharmaceutical Development, Jilin Agricultural University, Changchun, Jilin, China; 2 College of Life Sciences, Jilin Agricultural University, Changchun, Jilin, China; 3 Institute of Genomic Medicine, Wenzhou Medical College, Wenzhou, China; 4 Plant Germplasm and Genomics Center, Germplasm Bank of Wild Species (GBOWS), Kunming Institute of Botany, Chinese Academy of Sciences, Kunming, Yunnan, China; Michigan State University, United States of America

## Abstract

**Background:**

*Leymus chinensis* (Trin.) Tzvel. is a high saline-alkaline tolerant forage grass genus of the tribe Gramineae family, which also plays an important role in protection of natural environment. To date, little is known about the saline-alkaline tolerance of *L. chinensis* on the molecular level. To better understand the molecular mechanism of saline-alkaline tolerance in L. *chinensis*, 454 pyrosequencing was used for the transcriptome study.

**Results:**

We used Roche-454 massive parallel pyrosequencing technology to sequence two different cDNA libraries that were built from the two samples of control and under saline-alkaline treatment (optimal stress concentration-Hoagland solution with 100 mM NaCl and 200 mM NaHCO_3_). A total of 363,734 reads in control group and 526,267 reads in treatment group with an average length of 489 bp and 493 bp were obtained, respectively. The reads were assembled into 104,105 unigenes with MIRA sequence assemable software, among which, 73,665 unigenes were in control group, 88,016 unigenes in treatment group and 57,576 unigenes in both groups. According to the comparative expression analysis between the two groups with the threshold of “log2 Ratio ≥1”, there were 36,497 up-regulated unegenes and 18,218 down-regulated unigenes predicted to be the differentially expressed genes. After gene annotation and pathway enrichment analysis, most of them were involved in stress and tolerant function, signal transduction, energy production and conversion, and inorganic ion transport. Furthermore, 16 of these differentially expressed genes were selected for real-time PCR validation, and they were successfully confirmed with the results of 454 pyrosequencing.

**Conclusions:**

This work is the first time to study the transcriptome of *L. chinensis* under saline-alkaline treatment based on the 454-FLX massively parallel DNA sequencing platform. It also deepened studies on molecular mechanisms of saline-alkaline in *L. chinensis*, and constituted a database for future studies.

## Introduction

*Leymus chinensis* (Trin.) Tzvel., a perennial rhizome grass of the tribe Gramineae family with an allotetraploid species(2n = 4x = 28), naturally grew on alkaline-sodic soils in northern China [1], [2]. It's also an economically and ecologically important grass plant that contains many extremely valuable stress resistance genes [3]. Because of its high drought and saline–alkaline tolerance [4], [5], [6], *L. chinensis* plays an important role in the establishment of artificial grassland and in the protection of environment, which has received considerable attention in recent decades [7], [8]. Despite such advances, the genome of *L. chinensis* hasn't been published, little is known about its reference of genetic information on-line, and few studies have been reported on saline-alkaline of *L. chinensis* on molecular level. Therefore, the studies on molecular mechanisms of saline-alkaline in *L. chinensis* have far-reaching significance.

These years, a lot of studies have been reported on abiotic stresses of plants. The saline-alkaline stress is one of the main abiotic stresses, which is more seriously harmful than any single salt and alkaline stress on plants. Maybe salinity and alkalinity have a cooperative effect when they simultaneously stress on plants, which also had been demonstrated in *L. chinensis* and other species [9], [10], [11]. The mechanisms of abiotic stresses on plants are complex and diverse, even involve multiple complex physiological and metabolic pathways, which mostly include synthesis of extrusion and compartmentalization of sodium ions, response to abiotic stress, pathogen defense and adjustment of ion homeostasis [12], [13]. These mechanisms involve the expression of a cluster of genes and interaction among their gene products rather than individual genes, and the gene expression affected by many internal and external factors [14]. Therefore, the more comprehensive understanding of abiotic stress tolerance mechanisms need to be based on the gene expression level.

Over the past decades, the significant progress has been made in genome-wide gene expression profiling (GEP) by the development and application of differential display [15], as well as the large scale analysis of differential gene expression technology, such as cDNA libraries cloning technology [16], [17], [18], SAGE [19], Microarray technology [20], [21], and others. However, each of the above techniques has its disadvantages, such as high false positive rates, low level expression abundance, time-consuming and intensive labor [22]. As the first next-generation technology to reach the market, the development of the 454 Life Sciences (454; Branford, CT, USA; now Roche, Basel) sequencing platform (the 454 Sequencer) provides a compelling case study for the establishment of a new disruptive technology [23]. Moreover, 454 the 454-FLX massively parallel DNA sequencing platform is an effective next generation sequencing technology to better understand the transcriptome of unknown genome plant [24]. Meanwhile, massively parallel DNA sequencing platforms have become available which reduce the cost of DNA sequencing by over two orders of magnitude, making global transcriptome analysis inexpensive, and widespread [25]. Furthermore, a lot of studies on the comparative high throughput sequencing of plant transcriptome in many model and non model species, such us maize, grapevine, eucalyptus, olive genotype and cucumber flower have been reported [26], [27], [28], [29].

To gain a global view of the molecular mechanisms of saline-alkaline in *L. chinensis*, a transcriptome study on the two samples of control and saline-alkaline treatment (Hoagland solution with 100 mM NaCl and 200 mM NaHCO_3_) was performed to make a comparative gene expression analyses. Basing on barley, rice and wheat which closely relate to *L. chinensis* as references, we present a bioinformatic exploration, functional annotation, comparative analysis and real-time PCR validation of subset transcripts identified from significantly different expression of *L. chinensis*.

## Results

### Assay of Pro, SOD and MDA

**Figure 1:**
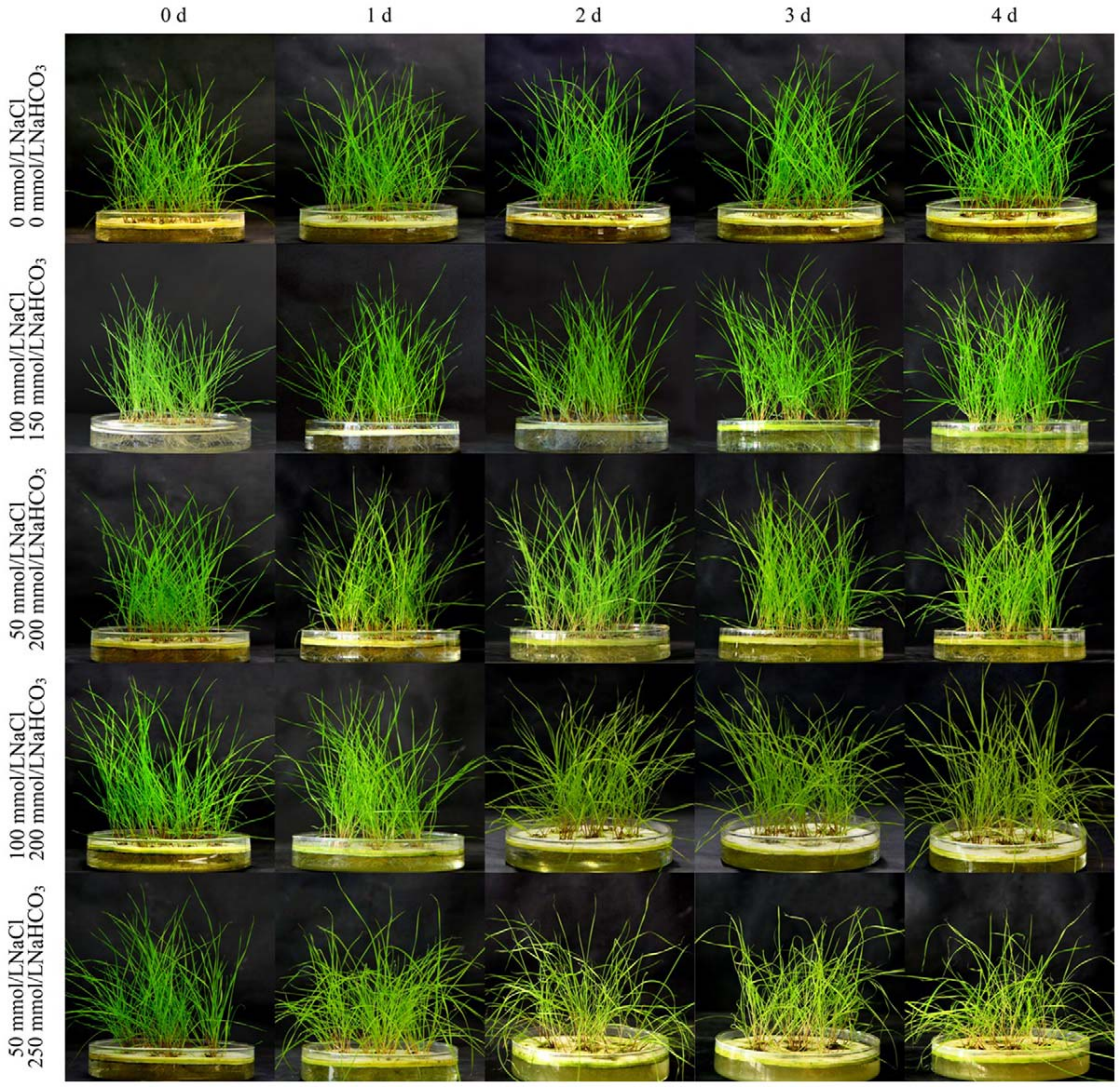
Photos, the growth status of *L. chinensis* under different NaCl/NaHCO_3_ treatment at different time. Abscissa indicates the different treatment time, ordinate indicates the different NaCl/NaHCO_3_ treatment.

**Figure 2:**
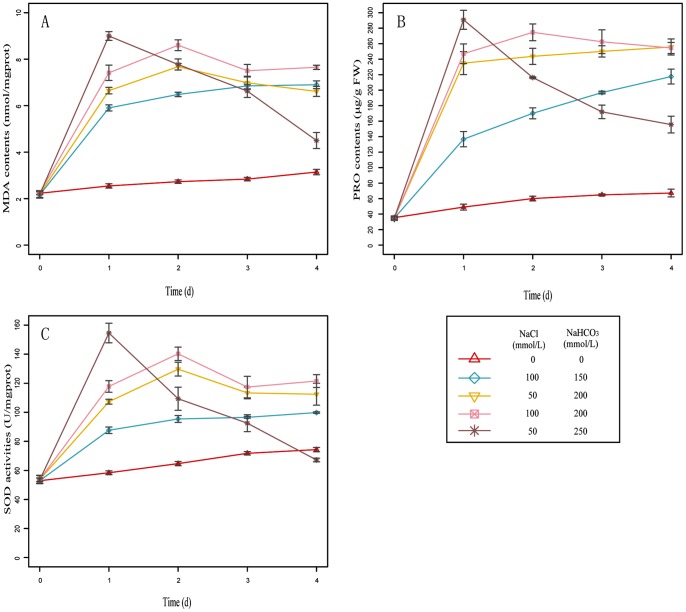
Polygram, the physiological activities changes of *L. chinensis* under different NaCl/NaHCO_3_ treatment at different time. Abscissa indicates the treatment time, ordinate indicates the physiological activities, and different color indicates different NaCl/NaHCO_3_ treatment. Data were obtained from three independent experiments and are means ± SE.

To explore the optimal saline-alkaline stress concentration which can be considered as the particular condition for more expression of transcripts, about one month old seedlings of *L. chinensis* were challenged to the stress treatments of various concentrations of NaCl and NaHCO_3_ (see *Materials and Methods*). The results showed that the plants grew normally under normal condition and the treatments of 100 mM NaCl+150 mM NaHCO_3_ and 50 mM NaCl+200 mM NaHCO_3_ during the whole period (Figure 1). The seedlings under the treatment of 100 mM NaCl and 200 mM NaHCO_3_ grew normally before the third day, and were slightly affected but could survive on the fourth day. While the treatment of 50 mM NaCl and 250 mM NaHCO_3_ seriously damaged the plants, and even caused the plants death after the second day. Furthmore, the proline (PRO) contents, superoxide dismutase (SOD) activities, and malondialdehyde (MDA) contents of all the collected samples were measured and showed in Figure 2. The results showed that PRO contents, SOD activities, and MDA contents reached the peak in one day, while dismounted rapidly afterwards at the stress concentration of 50 mM NaCl+250 mM NaHCO_3_, which indicated the physiological status of these plants were seriously damaged under this saline-alkaline stress condition. However, PRO contents, SOD activities, and MDA contents reached the peak in two days and kept the regular changing later at the stress concentration of 50 mM NaCl+200 mM NaHCO_3_ and 100 mM NaCl+200 mM NaHCO_3_. Moreover, the level of the PRO contents, SOD activities, and MDA contents under the treatment of 100 mM NaCl+200 mM NaHCO_3_ were higher than that of the treatment of 50 mM NaCl+200 mM NaHCO_3_ on each time point. Interestingly, PRO contents, SOD activities, and MDA contents haven't reached the peak until 4 days after stress treatments of 100 mM NaCl+150 mM NaHCO_3_ and under the normal condition, which indicated this saline-alkaline stress condition did not deeply induce the change of the plant's physiological status. These results, together with the plant growth status indicated that the optimal saline-alkaline stress condition was 100 mM NaCl+200 mM NaHCO_3_ for the second day, which was finally taken as the treatment group for the following experiments, sequencing and qRT-PCR.

### Sequencing output and assembly

**Figure 3:**
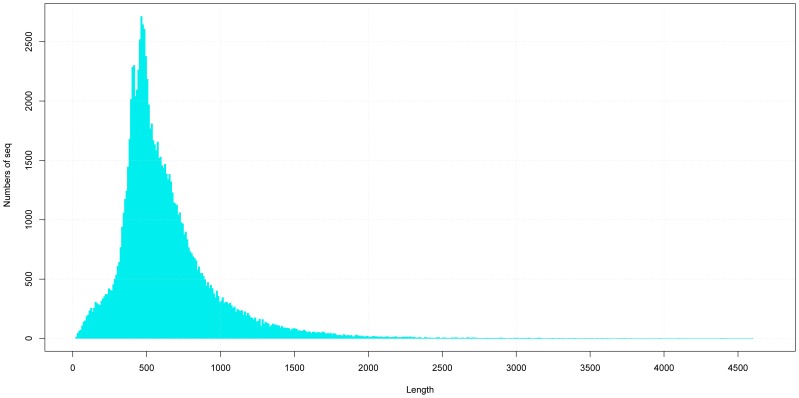
Histogram, the length distribution of assembled unigenes. The longest unigene is 4597 bp. The average length of unigenes is 630 bp.

**Figure 4:**
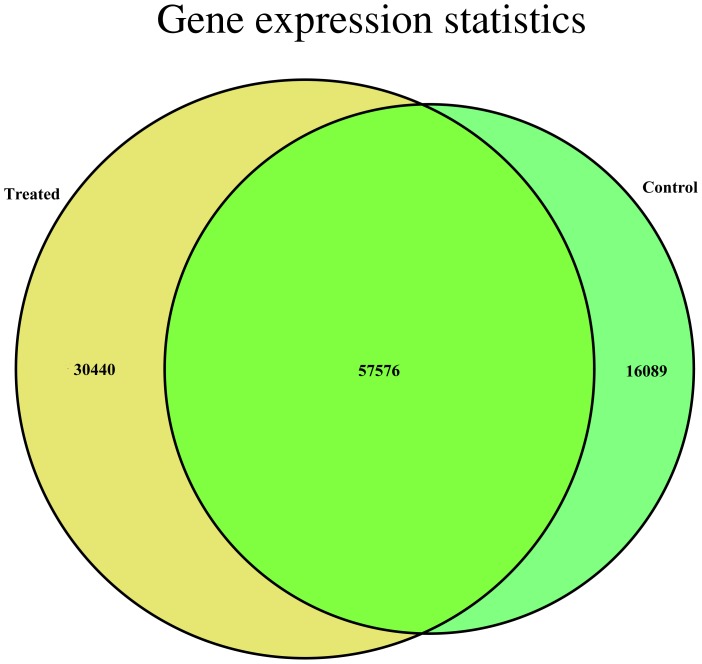
Venn diagram, the gene expression statistics of the two samples. (TIFF) The part of 16089 unigenes, 30440 unigenes and 57576 unigenes denotes the control group specific genes, the treated group specific genes, and the overlapped genes, respectively.

A total of 363,734 and 526,266 raw reads were generated in control and treatment samples by 454 sequencing, respectively (Table 1), and the raw reads data were submitted on the public database (Submission ID:SRA053207/Raw reads of *Leymus chinensis* transcriptomes). After filtering out low quality reads, short reads, contamination sequences and vector sequences, 362,664 and 525,198 clean reads were remained in control and treatment samples with average length of 489 bp and 493 bp for assembling. The two samples reads were totally assembled into 104,105 unigenes with an average length of 630 bp using MIRA program [30], and the longest one was 4,597 bp. The length distribution of assembled unigenes was presented in Figure 3. Among all the assembled unigenes, 73,665 unigenes were in control group, among them, 16,089 unigenes were unique for control group. 88,016 unigenes were in treatment group and concluding 36,440 unigenes unique for treatment group. 57,576 unigenes were shared by both groups (Figure 4).

**Table 1:** Sequencing, assembly and data statistics.

	Control	Treated
Raw reads	363734	526266
Low quality	936	900
Short reads (<50 bp)	3	1
Contamination sequences	119	136
Vector sequences	12	31
Clean reads	362664	525198
Average length	489	493

### Comparison analysis between control and treatment samples

**Figure 5:**
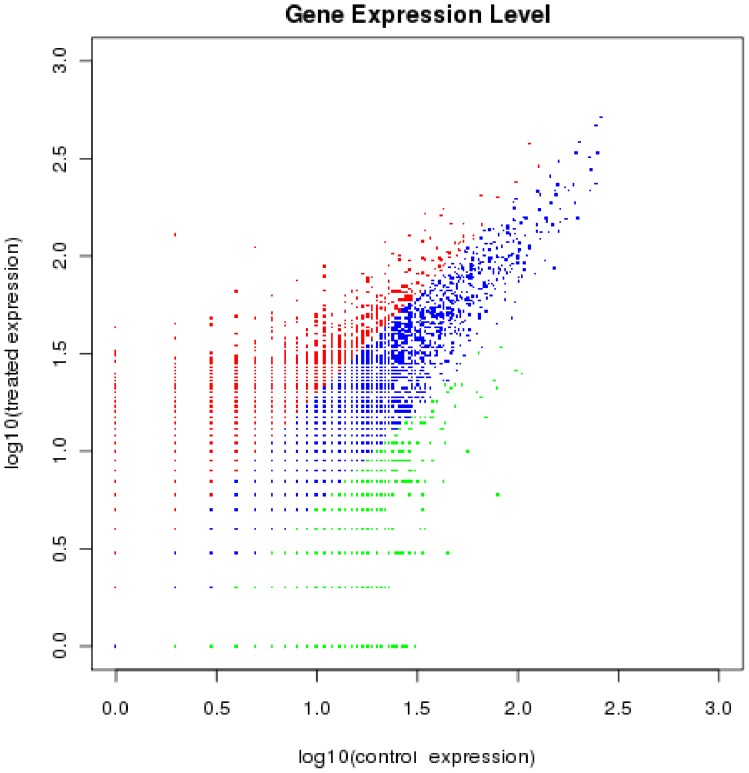
Scatter plot, the different expressed genes of the two samples. The blue dots that differed by less than two fold between the two libraries, defined “no difference in expression”, the red dots (50514) and green dots (26222) represented the up-regulated and down-regulated expressed genes.

Comparative gene expression analysis between the two groups was used for estimating the gene expression levels in response to saline-alkaline stress. The transcripts with different expression levels were shown in Figure 5: the blue dots defined as “no difference in expression” represented the unigenes which differed by less than two fold between the two libraries, with the threshold of “log2 Ratio ≥1”, there were 36,497 up-regulated unigenes (red dots) and 18,218 down-regulated unigenes (green dots) predicted to be the significantly differentially expressed genes (DEG) (Table S1 and Table S2).

### Functional annotation and classification

**Figure 6:**
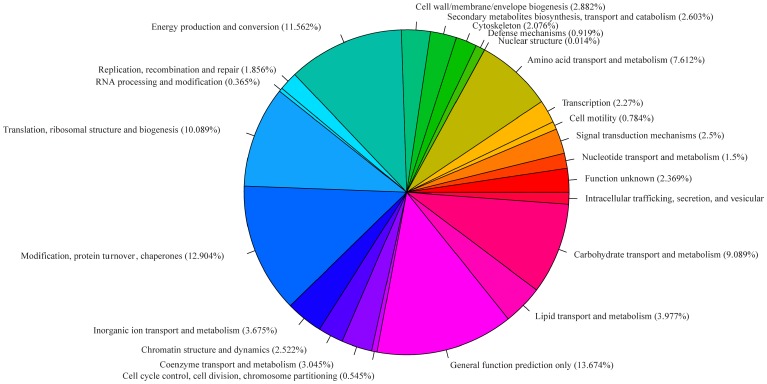
Paragraph, COG annotation and categorization of all unigenes. The unigenes were classified into different functional groups based on COG annotation.

The gene functional annotation were carried out and the results shown that 12.904% of unigenes were involved in modification, protein turnover, chapernes, 10.089% involved in translation, ribosomal structure and biogenesis, 11.562% involved in energy production and conversion. Other functional annotation results were shown in Figure 6. Furthermore, gene annotation based on the DEG was carried out. Multiple functional up- and down-regulated unigenes associated with the stress functions (Table S1 and Table S2). After stress functional filtering, we surprising found that the specific expressed more than 10 fold genes in control or treatment group were predicted to be closely related with the plant stress functions (Table 2).

**Table 2:** The specific expressed more than 10 fold genes related with the plant stress functions.

GeneID	Gene_length	log2 (treatment/control)	annotation
**Up-regulated genes**			
**Stress and tolerant function**			
GW_rep_c56407	768	13.425	salt stress-responsive protein [Triticum aestivum]
GW_rep_c55028	796	13.136	salt stress-responsive protein [Triticum aestivum]
GW_rep_c73217	676	12.773	salt stress-responsive protein [Triticum aestivum]
GW_rep_c57492	501	12.773	salt tolerant protein [Triticum aestivum]
GW_rep_c62560	524	12.551	salt tolerance protein [Zea mays]
GW_rep_c70666	667	12.551	salt stress-responsive protein [Triticum aestivum]
GW_rep_c82806	505	12.288	salt stress-responsive protein [Triticum aestivum]
GW_rep_c64764	635	12.288	salt stress-responsive protein [Triticum aestivum]
GW_rep_c74743	654	12.288	salt stress-responsive protein [Triticum aestivum]
GW_rep_c81453	319	12.288	salt stress-responsive protein [Triticum aestivum]
GW_rep_c59592	652	12.288	salt tolerance protein [Zea mays]
GW_rep_c74655	608	11.966	salt tolerant protein [Triticum aestivum]
GW_rep_c61447	642	11.966	salt tolerant protein [Triticum aestivum]
GW_rep_c83484	693	11.966	salt stress-responsive protein [Triticum aestivum]
GW_rep_c79794	417	11.551	salt stress-responsive protein [Triticum aestivum]
GW_rep_c82013	424	11.551	salt tolerant protein [Triticum aestivum]
GW_rep_c76556	438	11.551	salt stress-responsive protein [Triticum aestivum]
GW_rep_c88621	483	11.551	salt stress-responsive protein [Triticum aestivum]
GW_rep_c95199	519	11.551	salt tolerant protein [Triticum aestivum]
GW_rep_c66826	540	11.551	salt stress-responsive protein [Triticum aestivum]
GW_rep_c69291	590	11.551	salt tolerance protein [Zea mays]
GW_rep_c76526	723	11.551	salt tolerant protein [Triticum aestivum]
GW_rep_c61548	467	11.551	salt tolerant protein [Triticum aestivum]
GW_rep_c88723	561	11.551	salt tolerant protein [Triticum aestivum]
GW_rep_c91963	645	11.551	salt tolerant protein [Triticum aestivum]
GW_rep_c65200	843	11.551	salt tolerant protein [Triticum aestivum]
GW_rep_c62212	461	10.966	salt tolerant protein [Triticum aestivum]
GW_rep_c75697	274	10.966	salt tolerant protein [Triticum aestivum]
GW_rep_c72654	484	10.966	stress responsive protein [Triticum aestivum]
GW_rep_c75671	494	10.966	stress responsive protein [Triticum aestivum]
GW_rep_c96330	547	10.966	stress responsive protein [Triticum aestivum]
GW_rep_c87965	568	10.966	stress responsive protein [Zea mays]
GW_rep_c80875	583	10.966	stress-associated protein 8 [Oryza sativa Indica Group]
GW_rep_c89184	651	10.966	stress responsive protein [Triticum aestivum]
GW_rep_c84166	663	10.966	stress-associated protein 8 [Oryza sativa Indica Group]
GW_rep_c25561	471	10.966	stress-associated protein 8 [Oryza sativa Indica Group]
GW_rep_c75533	619	10.966	stress-associated protein 8 [Oryza sativa Indica Group]
GW_rep_c78874	631	10.966	stress-associated protein 8 [Oryza sativa Indica Group]
GW_rep_c66865	849	10.966	stress-associated protein 8 [Oryza sativa Indica Group]
GW_rep_c38686	518	10.966	stress responsive protein [Triticum aestivum]
GW_rep_c81211	692	10.966	stress responsive protein [Triticum aestivum]
GW_rep_c81415	338	10.966	universal stress protein 9303 [Hordeum vulgare subsp. vulgare]
GW_rep_c80789	356	10.966	universal stress protein 9308 [Hordeum vulgare subsp. vulgare]
GW_rep_c85023	393	10.966	universal stress protein 23267 [Hordeum vulgare subsp. vulgare]
**Energy production and conversion**			
GW_rep_c61665	537	12.773	ATP-citrate synthase, putative, expressed [Oryza sativa]
GW_rep_c64849	453	12.773	vacuolar ATP synthase subunit B [Zea mays]
GW_rep_c56023	903	12.551	ATP synthase beta subunit [Triticum aestivum]
GW_rep_c42513	417	12.551	ATP-citrate lyase B-1 [Arabidopsis lyrata subsp. lyrata]
GW_rep_c55569	401	12.551	vacuolar ATPase subunit G [Triticum aestivum]
GW_rep_c78632	466	11.966	vacuolar ATPase subunit G [Triticum aestivum]
GW_rep_c54695	591	11.966	vacuolar ATPase subunit F [Triticum aestivum]
GW_rep_c80539	399	11.966	vacuolar ATP synthetase subunit C [Aegilops tauschii]
GW_rep_c73093	514	11.551	ATP synthase beta subunit [Triticum aestivum]
GW_rep_c94788	765	11.551	ATP synthase subunit [Triticum aestivum]
GW_rep_c83052	744	11.551	vacuolar proton-ATPase subunit A [Triticum aestivum]
GW_rep_c76178	596	11.551	vacuolar ATPase subunit B1 [Triticum aestivum]
GW_rep_c54835	465	11.551	vacuolar proton ATPase subunit E [Triticum aestivum]
GW_rep_c59622	448	10.966	ATP synthase beta subunit [Triticum aestivum]
GW_rep_c89208	520	10.966	ATP synthase beta subunit [Triticum aestivum]
GW_rep_c80723	347	10.966	ATP synthase subunit [Triticum aestivum]
GW_rep_c82850	369	10.966	ATP synthase subunit [Triticum aestivum]
GW_rep_c78584	581	10.966	ATP synthase subunit [Triticum aestivum]
GW_rep_c95119	668	10.966	ATP synthase subunit [Triticum aestivum]
GW_rep_c75443	407	10.966	vacuolar ATPase subunit G [Triticum aestivum]
GW_rep_c88783	443	10.966	vacuolar H+-ATPase 16 kDa subunit c [Iris lactea var. chinensis]
GW_rep_c62927	530	10.966	vacuolar H+-ATPase 16 kDa subunit c [Iris lactea var. chinensis]
GW_rep_c48839	374	10.966	vacuolar proton-ATPase subunit A [Triticum aestivum]
GW_rep_c32981	353	10.966	vacuolar ATPase subunit F [Triticum aestivum]
GW_rep_c78047	382	10.966	vacuolar proton-inorganic pyrophosphatase [Hordeum vulgare]
GW_rep_c67872	411	10.966	vacuolar proton-inorganic pyrophosphatase [Hordeum vulgare]
GW_rep_c78888	593	10.966	vacuolar ATP synthetase subunit C [Aegilops tauschii]
GW_rep_c64609	642	10.966	vacuolar proton-ATPase subunit A [Triticum aestivum]
GW_rep_c87876	351	10.966	vacuolar ATPase subunit B1 [Triticum aestivum]
**Signal transduction mechanisms**			
GW_rep_c54463	845	14.214	calmodulin [Musa acuminata AAA Group]
GW_rep_c15343	673	11.966	calcium-dependent protein kinase [Triticum aestivum]
GW_rep_c89203	707	11.966	calmodulin-2 [Arabidopsis lyrata subsp. lyrata]
GW_rep_c61074	358	11.965	caltractin [Zea mays]
GW_rep_c23437	416	11.552	calcium-dependent protein kinase [Triticum aestivum]
GW_rep_c16881	433	11.551	caltractin [Zea mays]
GW_rep_c71382	556	11.551	calmodulin [Zea mays]
GW_rep_c70027	757	11.551	calmodulin-2 [Capsicum annuum]
GW_rep_c86210	382	10.966	calmodulin [Zea mays]
GW_rep_c55725	385	10.966	calmodulin [Zea mays]
GW_rep_c67900	414	10.966	calcium-dependent protein kinase [Triticum aestivum]
GW_rep_c86394	534	10.966	calmodulin2 [Zea mays]
GW_rep_c89734	556	10.966	calmodulin [Zea mays]
GW_rep_c90699	587	10.966	calmodulin2 [Zea mays]
**Inorganic ion transport and metabolism**			
GW_rep_c76883	767	12.773	transmembrane protein, putative, expressed [Oryza sativa]
GW_rep_c24417	397	12.288	plasma membrane H+-ATPase [Hordeum vulgare subsp. vulgare]
GW_rep_c60967	450	12.288	Ca2+/H+-exchanging protein [Hordeum vulgare subsp. vulgare]
GW_rep_c21310	629	11.966	plasma membrane H+-ATPase [Triticum aestivum]
GW_rep_c83286	552	11.966	Ca2+/H+-exchanging protein [Hordeum vulgare subsp. vulgare]
GW_rep_c96867	266	10.966	Na+/H+ antiporter precursor [Triticum aestivum]
GW_rep_c72884	454	10.966	vacuolar H+-pyrophosphatase [Triticum aestivum]
GW_rep_c69981	550	10.966	vacuolar proton-inorganic pyrophosphatase [Hordeum vulgare]
GW_rep_c89160	565	10.966	vacuolar proton-inorganic pyrophosphatase [Hordeum vulgare]
GW_rep_c61769	274	10.966	Na+/H+ antiporter [Puccinellia tenuiflora]
**Down-regulated genes**			
**Death**			
GW_rep_c105843	567	−10.966	DEAD/DEAH box helicase family protein [Oryza brachyantha]
GW_rep_c108908	420	−11.551	dead box ATP-dependent RNA helicase[Ricinus communis]
GW_rep_c12825	231	−12.551	DEAD-box ATPase-RNA-helicase [Triticum aestivum]
**Energy production and conversion**			
GW_rep_c107832	544	−10.966	peroxidase [Triticum aestivum]
GW_rep_c105379	480	−10.966	peroxidase 12 precursor [Zea mays]
GW_rep_c103913	491	−10.966	peroxidase 24 precursor [Zea mays]
GW_rep_c23688	441	−11.551	peroxidase 1 [Zea mays]
GW_rep_c101136	393	−11.966	peroxidase 4 [Triticum monococcum]
GW_rep_c101433	595	−12.288	peroxidase 16 precursor protein [Oryza sativa Indica Group]
**Damage**			
GW_rep_c67383	672	−10.966	wound/stress protein [Zea mays]
GW_rep_c84500	667	−11.551	wound/stress protein [Zea mays]
GW_rep_c55754	771	−12.965	wound/stress protein [Zea mays]
GW_rep_c67080	731	−13.287	wound/stress protein [Zea mays]
**Defence**			
GW_rep_c69512	506	−10.966	defender against death 1-like protein [Triticum aestivum]
GW_rep_c108309	651	−12.773	defender against death 1-like protein [Triticum aestivum]

### Pathway enrichment analysis of DEGs

Based on DEGs and annotation of KEGG, the biological pathways were evaluated by enrichment analysis of DEGs, a total of 120 pathways were up-regulated and 82 were down-regulated (Table S3, Table S4). With Q value<0.05 significantly enriched, the each first ten of up-regulated and down-regulated enriched pathways were reported in Table 3. Of which, calcium signaling pathway, oxidative phosphorylation, NHX antiporter were closely associated with stress function.

**Table 3:** The each first ten of up- and down-regulated enriched pathways.

KEGG Pathway	Pathway ID	DEGs Tested	Pvalue	Qvalue
**UP-regulated**				
Calcium signaling pathway	ko04020	287	6.36E-11	9.29E-09
Fatty acid biosynthesis	ko00071	238	2.05E-08	2.90E-06
Oxidative phosphorylation	ko00190	268	3.98E-08	2.90E-06
Flavonoid biosynthesis	ko00941	227	2.09E-07	2.00E-06
Peroxisome	ko04146	213	7.26E-07	3.53E-05
Cytokine-cytokine receptor interaction	ko04060	130	2.60E-06	3.53E-05
ABC transporters	ko02010	129	1.07903E-05	1.22E-05
Phenylpropanoid biosynthesis	ko00940	705	1.37E-05	4.46E-04
NHX antiporter	ko04260	302	4.70E-06	6.11E-03
RNA transport	ko03013	338	3.08E-02	6.04E-03
**Down-regulated**				
Glyoxylate and dicarboxylate metabolism	ko00630	659	1.24E-16	3.51E-14
Carbon fixation in photosynthetic organisms	ko00710	832	7.30E-15	1.03E-12
Metabolic pathways	ko01100	4088	1.24E-06	8.76E-05
Photosynthesis - antenna proteins	ko00196	827	0.000289561	1.17E-02
Chloroalkane and chloroalkene degradation	ko00625	100	0.000362629	1.28E-02
Carbohydrate digestion and absorption	ko04973	20	0.002161978	6.12E-02
Plant hormone signal transduction	ko04075	113	0.0267472	7.74E-02
Aldosterone-regulated sodium reabsorption	ko04960	29	0.006585828	3.55E-01
Caprolactam degradation	ko00930	30	0.00856093	2.86E-01
Tryptophan metabolism	ko00380	105	0.01302634	2.63E-01

### Validation of differentially expressed genes by qPCR

**Figure 7:**
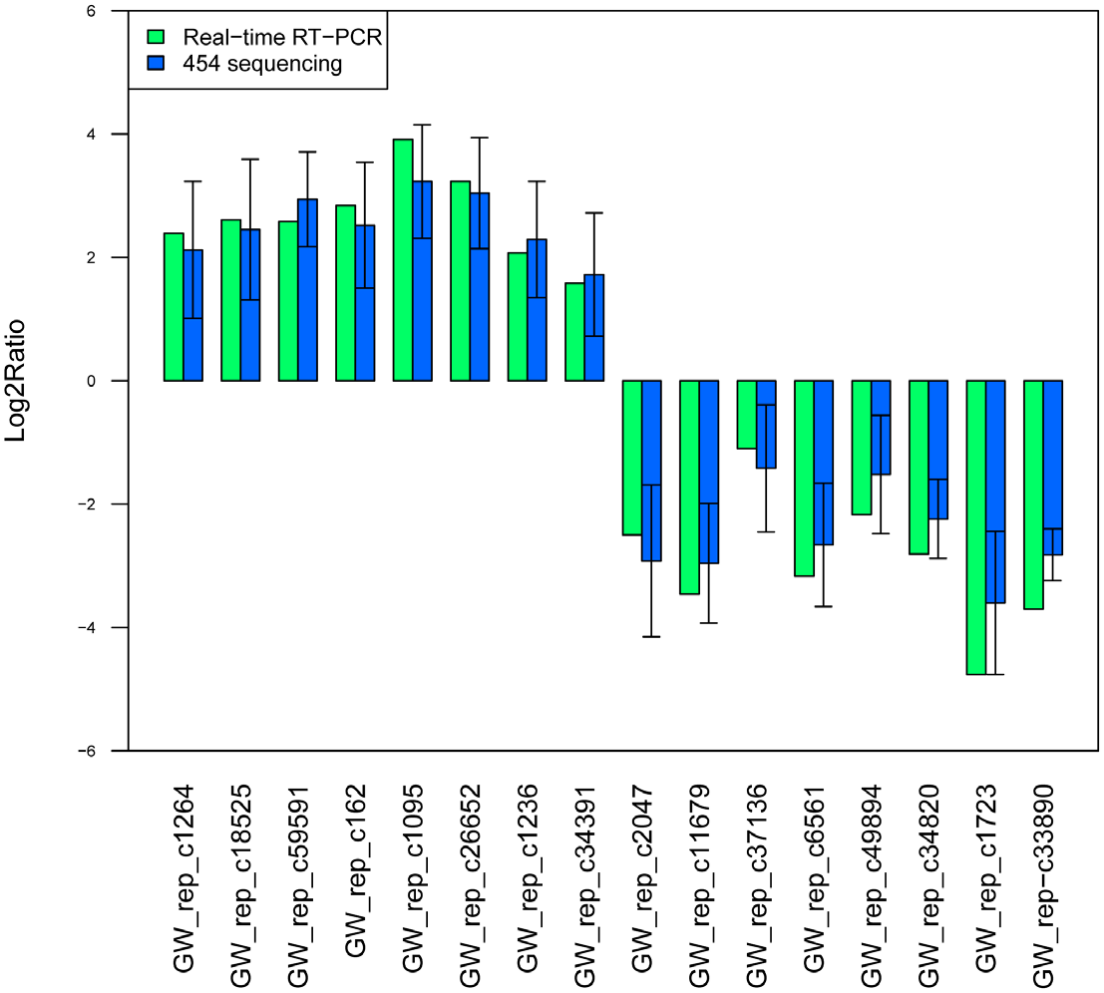
Histogram, Comparison of gene expression between qRT-PCR and 454 sequencing analysis. The qRT-PCR are presented as the mean values of three repeats.

Comparative expression analysis between the two groups, 16 unigenes were randomly selected from the differentially expressed genes and performed for further qRT-PCR validation. Among them, eight up-regulated unigenes (GW_rep_c1264, GW_rep_c59591, GW_rep_c1095, GW_rep_c1236, GW_rep_c34391, GW_rep_c26652, GW_rep_c162, GW_rep_c18525) were and eight down-regulated unigenes (GW_rep_c2047, GW_rep_c1723, GW_rep_c11679, GW_rep_c37136, GW_rep_c6561, GW_rep_c49894, GW_rep_c34820, GW_rep-c33890) were validated to correspond with the results of 454 sequencing (Figure 7, Text S1), the complete list of unigenes was listed in the Text S2, and the original RT figures were shown in the Text S3.

## Discussion

The main aim of this study was to better understand the molecular mechanism of saline-alkaline tolerance and to obtain a number of key genes and complex pathways that play a critical role in response to saline-alkaline in *L. chinensis*. Therefore, we started an effective way of the high throughput sequencing and comparative transcriptome analysis based on 454 sequencing platform under optimal saline-alkaline stress concentration (100 mM NaCl and 200 mM NaHCO_3_).

Large scale comparative transcriptome analysis based on next generation sequencing technology have effective way to study the initial molecular changes and complex pathways [26], [28]. Our study also mainly focused on comparative transcriptional level analysis of the differentially expressed unigenes between the two samples, which was also used for estimating gene expression levels in response to saline-alkaline stress. Among the 104,105 unigenes obtained, 73,665 unigenes were in control group, 88,016 unigenes were in treatment group and 57,576 unigenes were in both groups (Figure 4). The transcripts with different expression levels between the two samples were shown in Figure 5, With the threshold of “log2 Ratio ≥1”, the red dots (36497) and green dots (18218) represented the significantly differentially expressed genes(DEG), the proportion of up-regulated genes was higher than that of down-regulated genes.

Based on the annotation of these differentially expressed genes (Table S1 and Table S2), there were multiple functional up- and down-regulated unigenes predicted to be closely related with the plant stress functions. Those included stress and tolerant function (salt stress-responsive protein, salt tolerant protein, sodium/hydrogen exchanger, stress-associated protein, and universal stress protein), signal transduction (calmodulin, calcium-dependent protein kinase, caltractin), energy production and conversion (ATP-citrate synthase, vacuolar ATP synthase subunit B, ATP synthase beta subunit, ATP-citrate lyase, vacuolar ATPase subunit, vacuolar proton-ATPase subunit and vacuolar H^+^-ATPase), and inorganic ion transport (transmembrane protein, plasma membrane H+-ATPase, Ca^2+^/H^+^-exchanging protein, vacuolar H+-pyrophosphatase, vacuolar proton-inorganic pyrophosphatase, Na^+^/H^+^ antiporter). Other down-regulated unigenes were associated with Death (DEAD/DEAH box helicase family protein, DEAD-box ATPase-RNA-helicase), Energy production and conversion (peroxidase, oxidative stress), Damage (wound/stress protein), Defence (defender against death).

Pathway functional enrichment analyses were carried out, which revealed the most significantly affected pathways during the treatment process. Fortunately, calcium signaling, oxidative phosphorylation and NHX antiporter up-regulated pathways appeared in the first ten enriched pathways (Table 3); more detailed information of pathways was shown in Table S3 and Table S4. The plasma membrane is one of the main sites common to different stresses [31]. Calcium serves as a versatile messenger in many adaptation and developmental processes in plants [32]. Calcium binding proteins serve as sensor molecules to detect and transmit cellular calcium signals [33]. In higher plants, the main mechanism for Na^+^ extrusion is powered by the operation of the plasma membrane H^+^-ATPase and Ca^2+^-ATPase [34]. They use the energy of ATP hydrolysis generated from oxidative phosphorylation pathway to pump H^+^ and Na^+^ into the cell. This proton motive force generated by the H^+^-ATPase and Ca^2+^-ATPase operate Na^+^/H^+^ antiporter (NHX) and Na^+^/Ca^2+^ antiporter (NCX) of plasma membrane. It has been reported that the NCX removes a single calcium ion in exchange for the import of three sodium ions [35]. The operation of plasma membrane Na^+^/H^+^ antiporter has been obtained in different plant species [36], and Na^+^/H^+^ antiporter activity has been reported on the plasma membrane of tobacco, and wheat [37], [38], [39].

Due to the sensitivity of 454 pyrosequencing has been documented to be more sensitive for estimation of gene expression than traditional Sanger sequences [14], 454 pyrosequencing has the advantages of lower error rate, higher sequencing capacity and long read lengths (600 bp in average), which might be the best choice for the unknown genome plants [24]. However, it's relatively difficult and expensive may cause 454 pyrosequencing not widely accessible, in the longer term, the principles established by 454 sequencing might reduce cost further [40], [41]. In our study, in order to confirm the steady-state transcript level, 16 unigenes of the differentially expressed genes were selected for validation by Real-time RT-PCR. Although the results in gene expression didn't match perfectly to the results detected by 454 pyrosequencing method, the up- and down-regulated trends were closely similar (Figure 7). Furthermore, according to the description of these 16 validated genes, some of them were associated with plant stress functions, such as Energy production and conversion, Ca2+-binding protein, and defense mechanisms. Moreover, more genes will be validated in the future study.

### Conclusion

This is the first report of comprehensive transcriptome analysis and identification of differentially expressed genes of *L. chinensis* under saline-alkali stress based on the 454-FLX massively parallel DNA sequencing platform. The study showed that the responses to saline-alkali stress more serious than any single salt and alkali stress in *L. chinensis*, which had complex and diverse mechanisms, even involved multiple complex physiological and metabolic pathways. It will enrich our knowledge of the stress tolerance of *L. chinensis* at the molecular level and provide new insight to better understand the saline-alkali stress tolerance in other plants. All the data in our study will be of considerable archive for future studies.

## Materials and Methods

### Plants culture and treatment

Seeds of *L. chinensis* (Jisheng No. 4 Chinese Wildtye) with high saline-alkaline and drought resistance were obtained from Jilin Province Jisheng Wildrye Excellent Seed Station. After germinating in the dark for 72 h at 30°C, the well germinated seeds were evenly transferred into the hydroponic pots that contained Hoagland's nutrient solution in a artificial climate chamber with 15 h light (200 µEm-2s-1, 25°C) and 9 h dark (23°C), and with the relative humidity controlled at 75%. The nutrient solution was changed every 2 d. When seedlings of *L. chinensis* were about one month old, they were transferred into solutions supplemented with various concentrations of NaCl and NaHCO_3_ (100 mM NaCl+150 mM NaHCO_3_, 50 mM NaCl+200 mM NaHCO_3_, 100 mM NaCl+200 mM NaHCO_3_, 50 mM NaCl+250 mM NaHCO_3_) for 0 d, 1 d, 2 d, 3 d and 4 d. The control plants received no NaCl and Na_2_CO_3_ supplementation. The control and each treatment were biologically and temporally repeated in three independent and parallel experiments. The whole plants of the control and NaCl and NaHCO_3_ treatment were collected and then stored at −80°C until further use.

### Physiological Index measurement

UV-Vis spectrophotometers (Shimadzu, UV-2450) were used to measure the physiological indexes (SOD, PRO and MDA) [42], [43], [44], [45] of the stored samples with physiological assay kit (Nanjing Jiancheng Bioengineering Institute, Nanjing, China), respectively. All the processes were biologically and temporally repeated in three independent and parallel experiments.

### Total RNA extraction and cDNA synthesis

Total RNAs were extracted from the whole plants of the control and NaCl and NaHCO_3_ treatment using Trizol (Invitrogen) following the manufacturer's protocol. The quality of two total RNAs was checked using the NanoDrop Spectrometer (ND-1000 Spectrophotometer, Peqlab). The mRNAs were isolated from total RNAs using the PolyATtract® mRNA Isolation Systems kit (Promega, company) and condensed using Reasy RNA cleaning kit (QIAGEN, Germany), their concentration and purity were determined using the Agilent 2100 Bioanalyzer (RNA Nano Chip, Agilent). Using RNA Fragment reagent kit (Illumina, company) and Reasy RNA cleaning kit (QIAGEN, Germany) to fragment and retrieve the mRNA that has been condensed for 1 min. Then using random primer and MMLV to synthesize the first chain, and using DNA Polymerase I and RNase H to synthesize the second chain. Finally using Reasy RNA cleaning kit (QIAGEN, Germany) to retrieve cDNA, and using the Agilent 2100 Bioanalyzer to check the quality of cDNA. All procedures were applied according to the manufacturer's protocol.

### 454 sequencing and assembly

After linking with proprietary adapters sequentially, using GS-FLX platform with GS FLX Titanium kit to sequence approximately 10 ug cDNA from each of the two samples at sequencing company, a half-plate sequencing run was performed for each sample. The raw 454 sequence files in SFF format were base called using the Pyrobayes base Caller (Quinlan AR, 2008). Using Seqclean program (http://compbio.dfci.harvard.edu/tgi/software), LUCY program [46] and TagDust to clean the raw reads, including low quality reads, adaptor reads, short reads (<50 bp), polluted reads, hairpin structure reads and mosaic reads, all reads were assembled into unigenes using MIRA program [47]. Then all unigenes were used for all subsequent analysis.

### Comparison analysis between control and treatment samples

For the comparative expression analysis between the two samples, the number of clean reads in each sample was normalized to Tags (reads) Per Million (TPM) to normalized gene expression level. Significance of differential gene expression was determined using the R statistic and the resulting raw p values were corrected for multiple tests using the False Discovery Rate (FDR).Genes were deemed to be significantly differentially expressed with the threshold of “FDR<0.001” and “log2 Ratio ≥1”and an estimated absolute log2-fold change >1 in sequence counts across the two samples. Finally, pathway functional enrichment analysis was carried out from the differentially expressed genes. Pathway enrichment analysis based on Hypergeometric distribution was used to identify the significantly enriched functional classification or metabolic pathways in DEGs. The formula is:
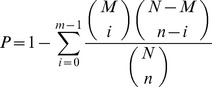



N is the total number of genes with KEGG functional annotations, and n is the number of DEGs in N. M is number of the gene with specific KEGG annotations, and m is the number of DEGs in M.

### Functional annotation and statistical classification

Functional annotation and classification of the unigenes was to predict possible highest similarity functions of unigenes and to do functional classification statistics using the following databases: NR protein database (NCBI), the gene ontology (GO), the Kyoto Encyclopedia of Genes and Genomes (KEGG) and the Clusters of Orthologous Groups database (COG; http://www.ncbi.nlm.nih.gov/COG/). Among them, all the unigenes were classified into different functional groups based on COG database and pathways were carried out by KEGG database.

### Quantitative real-time PCR (qRT-PCR) analysis

In order to verify the sequencing results, 16 of differentially expressed unigenes were randomly selected to confirm using quantitative real-time PCR. Primers specific for ubiquitin conjugating enzyme(UBC) (Forward: 5′-CGG AAA GGA TTG ACA GAT TGA-3′; Reverse: CTC AAT CTC GTG TGG CTG AA) were used for the normalization of reactions, which was used as an internal control [48]. Using the Premier 5.0 and Oligo 6 program to design primers with the length of 100 bp products or so, real-time PCR were performed using the SYBR *PremixExTaq*TM protocol (TaKaRa) on an Applied Biosystems 7500 Real-Time PCR System (Applied Biosystems, Foster City, CA, USA). All processes were performed in triplicate, and the average cycle thresholds (Ct) were used to determine fold-change. The relative quantification of gene expression was reported as a relative quantity (RQ) to the control value. The statistical package GraphPad Prism (GraphPad Software, Inc.) was calculated as 2− (ΔCt of treatment -ΔCt of control), which was used to analyze the data from all experiments [49].

## Supporting Information

Table S1 **The gene annotation of up-regulated genes.**
(XLS)

Table S2 **The gene annotation of down-regulated genes.**
(XLS)

Table S3 **The up-regulated enrichment pathways.**
(XLS)

Table S4 **The down-regulated enrichment pathways.**
(XLS)

Text S1 **Real-time PCR confirmation of differential expressed genes.**
(DOC)

Text S2 **The sequence list of confirmed unigenes.**
(DOC)

Text S3 **The original RT figures of 16 validated genes.**
(DOC)
