# Supplementary material for: Transcriptome Exploration in Leymus chinensis under Saline-Alkaline Treatment Using 454 Pyrosequencing
Source: PLoS One. 2013 Jan 24;8(1):e53632. doi: 10.1371/journal.pone.0053632 (PMC3554714; doi:10.1371/journal.pone.0053632)
Supplement: Text S1 — Real-time PCR confirmation of differential expressed genes. (DOC) [file pone.0053632.s005.doc]

**Table 4 Real-time PCR confirmation of differential expressed genes**

| GeneID | Gene_length | 454 sequence log2 (sample/control) | Real-timePCR log2 (sample/control) | primer(F) | primer(R) | Seq. Description |
| --- | --- | --- | --- | --- | --- | --- |
| GW_rep_c1264 | 116 | 2.39 | 2.12±1.11 | 5’-CACGGGCATAAGGAAACAT-3' | 5’-GTAGTGTTGCAAGTCCTGGGGTTCC-3' | Energy production and conversion |
| GW_rep_c18525 | 107 | 2.61 | 2.45±1.14 | 5'-GCT TGG TAA ACC CGT AAT C-3' | 5'-GTC AAC AAC GAG AAG AGC C-3'-3' | Amino acid transport and metabolism |
| GW_rep_c59591 | 118 | 2.58 | 2.94±0.77 | 5’-ACACCCGCTCCATCTCCTCC-3' | 5’-CGCCGCTCCTCCTCCAAGTC-3' | Ca2+-binding protein |
| GW_rep_c162 | 128 | 2.84 | 2.52±1.02 | 5'-TCG TGG TGG TTT GGA GGT G-3' | 5'-AAG CAA GGA GCA ACA TAA GC-3’ | Coenzyme transport and metabolism |
| GW_rep_c1095 | 115 | 3.91 | 3.23±0.92 | 5’-CGCTGGTTATGACCTTAT-3' | 5’-GATGCCCTGGTCCAGATT-3' | Amino acid transport and metabolism |
| GW_rep_c26652 | 138 | 3.23 | 3.04±0.90 | 5'-CTC ACG ACG GTC TAA TCC C-3’ | 5'-GGC AGC CAA GCG TTC ATA G-3' | cellular metabolic process |
| GW_rep_c1236 | 122 | 2.07 | 2.29±0.94 | 5’-TATCTCAACTGCTCGTCTTTC-3' | 5’-GCAATTCAGGCATACCTTTCA-3' | Cell wall/membrane/envelope biogenesis |
| GW_rep_c34391 | 127 | 1.58 | 1.72±1.00 | 5’-CAATGAGGTAGATCCTGGTGTT-3' | 5’-ATAAGCCTTTGACAGAATGGAA-3' | Defense mechanisms |
| GW_rep_c2047 | 124 | -2.5 | -2.92±1.23 | 5’-AACCCAACACCTAACTGACTA-3' | 5’-TTGACTTGCTGTCGCAATCTC-3' | RNA processing and modification |
| GW_rep_c11679 | 107 | -3.46 | -2.96±0.97 | 5’-ACCCTGTCTTACTTGCCACCG-3' | 5’-AACCCAACCTTGCTGAACTCG-3' | Energy production and conversion |
| GW_rep_c37136 | 104 | -1.1 | -1.42±1.03 | 5’-GCAGGCAGTATCTGATGTCC-3' | 5’-AGCAGTCGCCCTCAACCAAG-3' | Response regulator containing |
| GW_rep_c6561 | 123 | -3.17 | -2.66±1.00 | 5’-CCAACCTGCTTGCGGACTAAA-3' | 5’-TACGGTTACAAGGTCCTTCAT-3' | Predicted membrane protein |
| GW_rep_c49894 | 116 | -2.17 | -1.52±0.96 | 5’-CTTTGACATTGAGTGCCGTAT-3' | 5’-TGGAGGTCTGAGCCTTTCTAC-3' | Cell wall/membrane/envelope biogenesis |
| GW_rep_c34820 | 120 | -2.81 | -2.24±0.64 | 5’-GGCGACGAGGGGTTCATCCG-3' | 5’-ACCGCCCGCCCATCATCATC-3' | Posttranslational modification |
| GW_rep_c1723 | 136 | -4.76 | -3.60±1.16 | 5'-CTC AAA GGG TGA CAA CTG C-3'-3' | 5'-GTA CAA CCA CCT TGC GAT A-3' | Posttranslational modification |
| GW_rep-c33890 | 126 | -3.7 | -2.82±0.42 | 5’-ATTTTCCCATTCATTTTCATCG-3' | 5’-AACTTTGGTGTCTGCGGTTTCT-3' | F0F1-type ATP synthase, delta subunit |
