# Supplementary material for: Transcriptome Exploration in Leymus chinensis under Saline-Alkaline Treatment Using 454 Pyrosequencing
Source: PLoS One. 2013 Jan 24;8(1):e53632. doi: 10.1371/journal.pone.0053632 (PMC3554714; doi:10.1371/journal.pone.0053632)
Supplement: Text S2 — The sequence list of confirmed unigenes. (DOC) [file pone.0053632.s006.doc]

**The sequence list of confirmed unigenes:**

>GW_rep_c1264

CTGTCCCAACCCCAAGTGATTGCGAGATCACAAAATAACTCTTTGCTTCTTTTTTGTTTCCGGTTGTGGTGTACGAAAAAACCACACAGAAATTGTCGCAGATCACCATAGGATGTATGTCCAAATAGTAGCCTCTGTGCCTCTCTACTGCCACTTGAGTATATGTAAACCTTCATACCCTGAGATTGCCAGTTCTTCAGTGCCTCGGGAACATCCTCAAAAACAACTCCTTGCAGTTCTTTCCTTTCAAACCCAGTCCTCCATATATGACCCTGAAGTTGTTTCAATGATGTAATCTTCCGGTCTGCTTTGATCATTGCTTCAACATTAGCGACTAAAGAACTGATAACCTCTTCTTTGCCAGCATCATCCGGTGCAACTGGAGCAGCCCCAACAACTCCGATTTTAAGGTCTTCTTCTACTTGGATGCGTAAAAGTTTGATGTCTTCTTTGGTTTCCTCGGAATCGTATGTAGAAGTCAGATGCTTCCGCACATTATCACGGGCATAAGGAAACATAACATCAGTCACAAATGATATTGGTGTTGTTGTTCCTTCAATGTCAAGTACAACACAATGCTTTGATGATTTGGCTGCATGACATCCATTAGGAACCCCAGGACTTGCAACACTACGCAATCTTTTGGCACTGTTTATTGGGCCATGCTCAGGAGTTGTCCAGTCAATCCCTAACTGATACATCTTGATGGCAGCATCAAGAAGATAATGATAGCATTCAGCCTGTGTCTTGGCATTGATCCAGGAAACTCCGGAATGGCTGCGATTAGTATGTCTGACCCTTTCTGTTCTTCAAGACGTCCAATGAAAACTATAACAGGTCAGACATACTAATCGCAGCCATTCCGGAGTTTGTCGAGGAGAATGTTCAGATAATTGTTCTCGGCACAGGGAAGAAGAAAATGGAGGAGGAACTGATGCTGCTAGAAGTGAAGTACCCACAGAATGCCAGAGGCATAGCAAAATTCAATGTTCCATTGGCGCACATGATGTTCGCTGGGGCCGATTTCATAATTGTCCCAAGTAGGTTCGAGCCATGCGGCCTCATCCAACTGCAAGGGATGAGATATGGAGTGGTTCCCATCTGTTCATCCACCGGAGGACTCGTTGACACGGTGAGGGAAGGTGTCACCGGGTTCCACATGGGTTCGTTCAATGTCGAGTTTGAAACCGTCGATCCAGCAGATGTCGCGGCAGTCGCTTCGAACGTCACACGAGCTCTGAAACAGTACAAAACACCGTCGTTCCATGCAATGGTTCAGAATTGCATGGCGCAGGACCTATCTTGGAAGGGACCGGCAAAGAAGTGGGAGGAGGCTCTTCTTGGCCTAGGAGTCGAGGGAAGTCAGCCGGGCATTGAGGGCGAGGAGATCGCTCCACTTGCGAAACAAAATGTGGCCACTCCCTGAAATCTCCAAATATTAGGGAGTGTTCTAAGATTCGACGAAATAATGAGGTTGCTAAAGTTGAAGCTGCATTCATCTTGCCCTTTTCTCTACTACTCCATGTGTAACATTATACC

>GW_rep-c18525

CGCCCAGCTTCCTCTTCGAGTCCGTCGAGCAGGCCTCCGAGGGCACCAGTGTGGGGAGGTACAGCGTGGTCGGGGCCCAACCGGCCATGGAGATCGTGGCCAAGGCCAACCACGTGACGGTCATGGACCACGAGATGAGGACCAAGAAGGAGCAGTCCGCGTCTGATCCCATGAGCGTCCCAAGGGACATCATGGAGCAGTGGAACCCGCAGGTCACCGATGACGGCCTCCCTGACGCCTTTTGTGGAGGATGGGTTGGATTCTTCTCATATGATACGGTGCGTTATGTTGAGACAAAGAAGCTTCCGTTTTCCAAGGCGCCGGAGGATGATAGGAACCTTCCAGACATTCATCTAGGCCTCTACAATGACGTAGTCATCTTTGATCATGTTGAAAAGAAAACACATGTTATTCATTGGGTGAGGTTGGACTGCTATCACTCAATTGATGAAGCATATGAAGATGGGAAAAATCGTTGGAGCTTTGTTATTATAAATGGTGCTTTTATTAACTCACAATATAACATCAAGGAGTTACAAAGCATAATGAGCATACACCCGACCTTTGTTTGATAAGGATGCACAAACCCAACAACAATTCACGCACACGAGATTCTTTTATTAGCAAACATTAATTGTGCAGCTGACCAATTTTATTGCGCGACACTCCATGACGGCTGGACCATCAGGAAGCGAGCGTCGTGGGGAAGCAACTGTCGTGCACGCAGCTGAGCACGTCGCGATAATCCCTGTGGATGGTGAAGGAGTCGTAGACCTTGCCGTTGCTGCTCTTCATGTACTCGAACAAAAGCGATGAGTGGTTGAAGGCCGTCAGCTTGGTAAACCCGTAATCGTGATCCCTGAATATGCTCCACTTGGGGATCGCAGTGGTGTAGTCCGACAGGTGGCTGCCGCCGCCGCCGGCCACGACGAAGATGGTCCCGTTCATGGTGCCTGAGTAGTGGCTCTTCTCGTTGTTGACACACTGGCTCTGGTAGAGCGGGCATGTGCGCTCGTAATTATGGACGTGGCCGAAGTAGGCAATGTCGACGCGGTACCGCTGCCATAATTTCTGCAGGCTCTCCCGGCCCTCGGGCTCCTCGAAGGAGCCCTGTTTGGCGTACCATGAGTTGGAGGAGTAACCGAGCACCCGGTGCGCCGTGAAGATTAGCCACGGCTGGTGCTTGCGGTCCACCGTGGAGAGGCACTCCTCGATGAACTTGTACTGTGGAGTCCCCTCCCGCCAGTCGTGCTCCGAGTCCGCCACGCAGAACCGGAACATCCCGTAGTCCACCTTGTACCAAAAGTTTGCTCTGTTTTCGGCTGGGTAGTAGTACATGGTCTCGGCCGGCACACCGCATTCGCCGCCGGAGTCCTTGACGTCGAAAAATCCACCAGTGTTGGGCCAGTCCCTCTCGTGGTTGCCGCTTGCAACCATGTAGGGCTTCTTGGCGCTGATTGGGGCGACCTGTGCGGTGAATTGATCCCACTGGGAGAGGTACCCGTTGGCGTAGGGCATGTCGCCAATGTGGAAGACGATGTCGTAGTTGTCCAAATCTTCAATCAGCCTGTCCGTCGTGTTGAGTGACCCCGGCTGGTAGTTGGCAAATTCGTTTGATCCGTCCCTCTCTGCCTTTCCCATGTCACCAAAGACGATGATGCGCTGCAGCGAGTTCTGCCCGGGGGTTGGTGGCGCCCGGAAAGTGTAGGACTTGGCCCACACCACGGTTCCGTCGGAGAGCTCATGCCCAATCTTATAGAAGTACTCTTTGTTGGGCCACAGGTTCCTCATGAACGCCGTGTGGATGAACCCAGGATCTCTCCACCCAACAGTTCGCGCCGGCTCGCCGCACATGCTCCCGCGGTTGAAGGTCAGTGTCCCGGCGGGAGTGCGCGTGCCGTCGGAGCCGACCATGCCCCACTCCACGAAGGGGTAGGCCTCGCTGACGTCGTAGCCGCTGGTCCAGGTCACGGTCATCTCGTCGTGGGTCTTGCCCTGCGCCAGCCGCGGGAACACCGGCGCCTTGGGGTTCTTGAACACCACCGGCTTCGACACCGCCACCAGCTTCGGGTTTTCGAGGCCGCTGGTGAAGAGGGCGAAGGAGAAGTCGGCGCGCTGGTTGATGAGCTGGAGCCGGATGGTACCCTTGCCCCCTTTGAGGTAGTTCGCCGAGTAGTTGGCGTACTGATACTTGATAGGCGCCGTGCAGAGCAGTGGCTCGGCGGGGTACCTCTCAGGGTTAGGGCACGTGCCCGAGATGAAATCGGCGGGGGAGAAGACGGCGATCCAGTCATCGGGGG

AGGGGTTTTCCGAACCGTATTTGACGGTGACCCATACAGTGTCTTCGTCCTGGTTGCCGAGCAGCGCCGGCGTCGCCCGCACGTACGCGGAGGAGCGGTGCAGGTCAAGGTGAACGGTGGCCTTGTGGATGGCGATCTTGGACAGCGGCTGGATCCCCTGGGCCGGCGACGCGCTCACCGTCGCCGCCGCTGCCGCCTGCGCCTGCCTCCCGGCCCCCGTCTCCGCCATCCGGAAGGACATTGGTTTTGCTGCACCCATTATRTGCAGGAGAACTGTTCAAGGCCGGCATTTGATATCAGATGATAATGGTTATGTATGTTCTGCCCTTTCGATCGATCCATGGTCTCGCTGCTGCCCACGAACWGGAGCACGCTTCTCCTGTCAAGGCTGCAAGCTTGATTTACAGTGCTGCAATTCTTATGAGTATTGTGTTTCTTGCTGCTTGAATCCTTCTAGGACCAAGGAAGGAGATGTCGTTAAGTTGAAGGTAGCTAAGCCAGTTACTGCTGGAACTTACACCAATATCTTCGATTTCTGCATGGGAAGATGCCGCCATAGTTCTGCAAGCGTGGTCCATGAAAATGCATATTTGAGTGATTTCCATCATTGCTTCCTGGTGCAGCAAAATTCATCGGGATCAACTGAATCTAATTTTGGATCAAGGCTGGATGGCATTAACGTCATTTTGGGAAGGCAGGGGGAATCGTGTAGTTCTGCATGCAGAGCAAAGGACAATCATGTGTTCCAAGCAGGCTTTCTGAGTTGAACAAATGTCAGATTTTGCAGAAATATATGAGATGCAAAAATGGTTGCTTTCCCAGTCTTGGGCCCGATCAACCTAGTTCCGACGTTGTACGTAGAAGTCTTCCTAAAAAAGTTTGAAAATCCTAGGAAGCGTTGTCTTGTACATGCAGATGGATGACCGCCT

>GW_rep_c59591

CTTGCCTCCCACGACTAAAGAAACAAAGAATCTGGTACTAACGTACGCTCTAATGGCCACCCTTTAATCCTTCAATTATGTACACTTAATTCGCACTTAATTTGCACCGCACGCGAGAGGAAAGGAGGAAGGAGTGGACGAAATCTGTCCGCGGACGATGGGATGGATCAGGCCATCTTGGCGGCGAACCCGGCGGCCATCATGACCTTGAACTCGTCGAAGGAGATGAGCCCGTCGCCGTTCTTGTCGACGCCCTCGATCATGCGGCGGCACTGCTGCACGGTGGCCTTCTCGCCGAGGCCGTGGAGCACGCGCGCCAGCTCGGCGGCGGAGATGGCGCCGCTGCCGTCCGCGTCGAACACCTTGAAGGCGAGCCGCAGGTCCTCCTCGTCGTCGCCCGCCGCGGTGGCGTTGAGCGCCGCGAACTCCTCCAGGCTGATGAAGCCGTCGCCGTCGGCGTCCGCCTCCGCCATCATGCGGGACAGCTCGTCGTCGGTGACCGCGTGGCCCAGGCTCTCGAACAGCGCCGCCAGCTCCGGCCGCGAGATCCGCCCGTCGCCGTTGGCGTCGAACTTGCGGAACACCCGCTCCATCTCCTCCTCCGCCGACCGCGCCGGGGACCCCGCGCCCGACGCCGGCCGGTTCCCCTCCTCCTGGGGCTGGGTCTGCGGCGGCGGCGACTTGGAGGAGGAGCGGCGGCGGAACAGGGACGGCATCTTGATCTTGCCCATCGTCGTCGTCTACTTAGCGGCGCTAACAACGAAGAAGAAACTCTTGTCTTGGATTGG

>GW_rep_c162

ACCACCAAACCGCGGGCCGCATCCCGCGCCGCCCACCGGCGGCAAAGCCACCGSCGSWAAGCCACCGCCGCGACTATAGCCCAAGCAACCGGCACACACCAACCCCCACCCAGCCATGGCGTCCTCTCTCCTCACCACCCCGTCCCAGAACCTCGCCCTCACGCCCGCCGCGGCGCGCGCCCGCAGGTCCTCGCCTGCGGCGGCCCAGGTGTCCTTCTCCTCTCCCCGCCTCCCCGGTCGCCGGCCCTGCGCTTGCGCGCGTCCGTCGCGATCGAGAAGGAGGTGCCGGAGAACGAGGCGCCGACGACGTTCCTGCGGGAGGACGGCTCGGGGGCCGGGTCCGGGTCTGTGCGCGAGCGGTTCGAGGGCATGATCCGGCGGGTGCAGGGGGAGATCTGCGCCGCGCTCGAGGAGGCCGACGGCAGCGGGAAGCGGTTCGTGGAGGACGTGTGGTCGCGCCCCGGGGGCGGCGGGGGCATCAGCCGGGTGCTCCAGGACGGGAACGTGTTCGAGAAGGCCGGTGTGAACGTGTCCGTCGTGTACGGGGTCATGCCCCCGGACGCCTACCGCGCGGCCAAGGGGGCTGCGAAGAATGGGGCGGCCGATGGGCACAAGGCTGGGCCTGTGCCCTTCTTTGCCGCCGGCATTAGCTCGGTTCTTCATCCCAAGAATCCGTTTGCTCCAACATTGCATTTCAACTATCGTTATTTTGAAACAGATGCTCCAAAAGATGTACCTGGTGCACCAAGATCGTGGTGGTTTGGAGGTGGTACTGATTTGACTCCTTCCTATCTCATTGAAGAGGATGTGAAGCATTTTCATTTTACTTTTCAACACTGGGCATCCTACTGTTTCATGATATATATAGTATTTTGGCCTTCTGCTTATGTTGCTCCTTGCTTACATGTGTATATTAGTACTTAGATCATGCACCTTTTCCAAAAGAATATCTCCTTCCAACTGCTGAGCTTGGAATAACACGAAAAGTTAAATTTTGAGATAAATCTAARGTATTCAGTAATAGTAAGTTAGTGTATTCACCATATCTACTTCCTCTGGATAAAGTAATGACCTAATTACTTTTGACCATGTTGATGATCTCAACAAGGGAGATTATAAAGTAGTTTTACGACCACTATAATTGGACCTTTGTGATTATAACTATGT

>GW_rep_c1095

GAGGATTATTCTACAAAAATTGCTCTACAGCAGGGATCCTCTACTCATTGCAAAGGAAGATCCTTGTGGGTGTACACAACTCCCTTGTCGCCGTTCACTACTTGTCCACTCCCTCGCAGAATGCACCGAGTGGTTAAAAGACCGTCGACACCAACAGGTCCACGAGCATGTATGCGCCCTGTACTTATGCCAACCTCTGCACCTAGACCGAAGCGAGTCCCATCACAGAACCTTGTGCTTGCATTATGGAACACAGCAGCACTGTCAACTTGTTGTAGAAAAGTATCTGCTGATTTCGTATCAGTTGTGATAATACAATCTGTGTGTGCACTTCCATAACGATTTATATGGTCAATCGCTGACTGTACATCATCAACAAATTCGAGGGTGCATGCCATTGAGCTATACTCATGATGAAATGAATCTACCTTTGGTACTTTCAATGTGTCATGTGCGACAGGCCCACCATAAATAACAACTCCTTCTTTCGCAAGTTCCATCAATAAATCATCAAGACCCTCTGTCTTGTTCAAATCTTTATGAACAAGTARTGTTTCCATAGCATTACACGCTGCAGGATAATCAACCTTTGCATCCAACACGATACGTTTTGCCATATCCATGTCAGCTGATTTATCAATATAAACATGGCAGATACCCTCAGCATGACCAAGAACGGGAATCTTGGTTTGTGCTTTGATTTGAGAAACAAGCCTGTTACTGCCTCTAGGAATAACAAGATCAATCACATCGTCAAGCTTTAGAAGATCAGCGATTTCATCTTTGCTTTTTACAAGGCCAATAAGCTTTTTACCAACAACATCTGGAATCACGCTGGTTATGACCTTATGCAATATTGTGTTTGATCTCATAGCTTCTTTTCCTCCTTTCAGCAGAAGACCATTTCCACTTCGGATTGCTAGAGCTGCAATCTGGACCAGGGCATCAGGACGAGACTCAAAAATAATTAGGAGAACACCCAATGGGCAGTACATCTTCTCAAAAACTAAATCCTTAGCAACCTCTGTTTTTTTCAGTGTATGTGAGATAGGGTCCTCCATGTCCGCAATTGCACGAATCGATTCAGCAAGGCTTGTTATCTTTCCTGCCTTCAGGGTCATCCTAGCAACCAAGGATTTCTCGTAACCTGAATCTTGTGCCTCAGCTAGATCAGCTTCATTCTCGGATATAATTAAATCCACATTTGCTTCCAGAGCACCGGCAATATCTAGCAAAATCTTCTTACGCTCCTCCGATGACAAATTCTGGAGATGCCGTGAGCAATCTTTTGCAGCAACTGCCATCTCACGGGTTGTCACCTCCTTGGTACAATCCCAGATATTTGCTTCATTATGGAAAAGTGTACCAATTTTCTCTCCTCGCATAATCTTAATAATGCTATCTGTTACAAATCCACTTGCAATTACAGCAGGTACGCCCTTTGATGCAGCAGTAACAGCAGCTGCCACTTTAGCTTGCATTCCACCTCTTCCCACACGAGACTTCTCCCCAAAAATTAATTAGCTTCCCGTGTTTTTCATTGATGTATGTGTGGATAATCTTTGATTGAGGATCGCTCGGTGGACCACTATAGAGTCCCTCCACATCACTAAGCATGATAAGAAGATCAGCATCCAGTTCTTTCGCCAACAGCGTCGCCAAACTGTCGTTATCCCAGAATATACCGGATGAATCCTCGTATGGCGCTCTCCTCGTACTGATAGCATCGTTCTCGTTGAACACCGGTATCACCTTAAGATCTAACAGGGAGACAACAGTCTCACGAAGCTGGTGCCCAAAACTTGGATCCCGGAAATCACGATCGGTAACAAGAAGCTGAGACGATGTTACATCGAGTTGGCTAAACAGTGTATCGTAGATAGCCATTAGGCCACTCTGGCCGACGGCAGCACAGGCCTTCCCGTCCAGGTCCAACTGTGGGTTCTGCAGATCGGCGAAACTGCTGTTGATAAGCTTTCGGTACTTGAGCCTCTGCCTTCCGACGCCAACGGCGCCGGAGGTGACCAGAATCACCTCATACCCCTGGAAATTAAGCTCCTTCACCTGCTCGCAGAGAGCTCCGAGCCTGCCCATGGCCAGTCGGCCATTCTGCCCGGTGACAACAGCGGTGCCGACCTTGACGACGATGCGCTTGACGTCCCTGACGAAGCAGCGGGTGGAGTCGCTGTTCTCGAGGTCCGCCGCCGCAACGGCCCCTCCGATGCCTCCCCTGCCCATTCTCCGCTTCGCCGGGGCGTGGCGACCCGCGGGCGCTCGCTCTGG

>GW_rep_c26652

TGGCAGCCTCCATGATCACCTCGCCGATTGTGGCACCGACGAGCCTGCCGTCCCTCTCCCGCCGGGGCTCCTCCTTCGCCGTCGTCTGCAGCGGTGGCAAGAAGATCAAGACCGACAAGCCCCTCGGAATCGGAGGCGGTTTGACAGTCGACATCGACACGAACGGCACATTAGTTGATCCACTACATGGAGCGCCCGATGGATGCAGAACAGCTATGGCCGGACAAGGTTCTAGCCGATGATCTGGTCGAGGACGCCGGTGTTCTTGAGGCCGAGCACGATGCCGACGCCCATGATGTGGCCGACGGCGCCGCATGCGAGCGTGTCGGCGAGCGTGAAGCCGGCCGGGTCGCCCGTCTGCAGCCCGGAGTCGCGCGCCTCCAGCTTGAGCCCCGCCGTCGCCTTCCGGTTCGCCGACGGCGCCAGCCCGAACCGCCCGGCGAACAGCATCAGGGTCGTCGTCGTCACCATGATGAGGTTGGTGGAGGAGCCGATGTAGTCGCAGCGGAWGCCCTGGGACCTCTTCCTCAAGGACGGCAGCGTCGCCATGGACCTGGGCGAGGAGTAGGTCCTGAGGCCGTGGAACTGCGGCACGGAGGTCATGGCGGAGAGCTGGGAAGCCATGCGRCGGATTCGTCTGCAAAGGATTCAGCCCACCGCCCGTTGGGAAGGGAGCTTCGAGGCGGCCGGCCGCGGCACATCGGCCGGACCGGCTTAGCCAATGGCACGGGCCCTTGGGGGCGCAAGCGCCCCTAACGTGGGTCGGGGCGGGCGGCGGGCGCAGGCGTCGCATGCTAGCTTGGATTCTGACTTAGAGGCGTTCAGTCATAATCCGACACACGGTAGCTTCGCGCCACTGGCTTTTCAACCAAGCGCGATGACCAATTGTGTGAATCAACGGTTCCTCTCGTACTAGGTTGAATTACTATCGCGACACTGTCATCAGTAGGGTAAAACAACCTGTCTCACGACGGTCTAATCCCAGCTCACGTTCCCTATTGGTGGGTGAACAATCCAACACTTGGTGAATTCTGCTTCACAATGATAGGAAGAGCCGACATCGAAGGATCAAAAAGCAACGTCGCTATGAACGCTTGGCTGCCACAAGCCAGTTATCCCTGTGGTAACTTTTCTGACACCTCTAGCTTCAAACTCCGAAGATCTAAAGGATCGATAGGAGTCGTGGGAGGCAAGGC

>GW_rep_c1236

GTGACAACTCTGCACCTATGTACTGACTGTTACTGCCCTTGTATGGAATTCCACTACAGTGTCAAATTAAAATGCACTGCACTCTGTGCTTCAGAGTGCCACTAGTTCGATTCAGCAGCTGCAAATAGTTGCTTCCATTTCTCCTTGTCGCTCCCAGGTCCTTCGCCCTGCACCACGTCATTTGCCATATCAGTCGGAAGGAAAAGAAATTTCAATTGGTAACTAGGAAAATAAACAACGATTCATTTCGAGCAAGGTCTGTTTGATGTGTAACGCTAGTTTGCTCACAAAATTCACGCCATGAGTCCATCTGAATACCGGGATAAAAATAAATAGATAATGCAAGCAAAGGTTTAAAGCACGAGTTAACATCGTGTCCGAAGCATGCATGAGCACCTTCACCGAACTAATCTCGTATATTTTTCCTGGGTAGATTCAATATCCAAAGCTTGGATACATGCTTCTGCCACCACCAATCTGCTCGCTTCTCCCACAAGCTTGTCACCTTGTCCTATCTCAACTGCTCGTCTTTCTCCAGCTGTAGCCTTAACAAGTGTGTTCAGGTCATAGGAAGTGTAGGGCCCATCTGTTAATCTCCCCGGCCTGATGATGGTGAAAGGTATGCCTGAATTGCGGACAAAGTCCTCTGCCATCTTCTTGTATTTAAGCACACCAAAGAGGTTCATGATACTCCATGGTATTTCATTGTATTTTGTAACACCAATCGATGACACCAAAACCAGTCTCTTGATCGTCTGTGGCATGGCACTCACAAAATTTCGGACGCCATCCCAATCTACACGTTCAGGAGTGTTATCCCCATCCCAGCGTTTTGATGGAAATGCTGTAGTCCCAGTAGTACAGATCACATGTGTAACTCCCTCAAACATTTCTGGATTCAAATCGTCAGCATTTCTCGTGTCCGCTTCATAAACCTGCAAAACACTCTCATCCTGCTTGCCAAATAAGGACGCTGCCTTTTCAGGGTTTCTTAGGAGCAGCCTTGTCTTAATTTTCCTGCCCAGCAAAGATGCTACCACCAACTGACCTACGCCGCCGGTTCCGCCGACGACGAGGACCAGCCTCGAGGAGGCCTCGGCCTTTTCCTGCTGCGCGGTCCTGGGCTCCACTGCCGCCGCGAGCCGCCACCCACCGCGACCCGACGCGGCGAGTCTCGAGGAAAAGGGAAGCGAGCAAGACCGCAGCACCGCTGTGCCGCCTGGCTGCGCGAAGACGGGCGGCTGGCCGTGTTTGCACGTTAGGTGGCGCGACGATGAGACCGCGAGCGCCGGGCTAAGGTGAGCCGTCGTRTGTGGAGGCAAGG

>GW_rep_c34391

TGATGAGGATGTTCTTACCGAGGAAAATCTAGTAAAAGAACAAGCTGCAGGCAATGAGGTAGATCCTGGTGTTGCGGTTCAAATACGTGGCTTGCGGAAAACTTAATCCAGGAAGTTTTAATTATGGGTTGCTGCAAGTGCAGAACAACTAAGCCATTCCATTCTGTCAAAGGCTTATGGGTGGAACCTTGAGAAGGACCAGCTGTTTTGTTCTTCTTGGGCCAAATGGAGCTGGTAAAACAACTACGATCAGTTGCCTGACTGGAATCACACCAATTACAGGCGGCGATGCATTGATATATGGTCATTCCGTTCGAAGCACTGCGGGTATGTCTAATATTCGCAGAATGATTGGAGTCTGTCCACAGTTTGACATCCTGTGGGATGCATTGACGGCTAAGGAGCACATGGAGTTGTTTGCCAGCATCAAGGGGTTGCCACCATCAACAATCAAGTCGGTAGCAGAACAGTCACTAGCCCAAGTGAAGCTCAGCCAGGCAGCTAATGTTAGAGCAGGTAGCTATAGTGGTGGAATGAAACGGCGGTTAAGTGTTGCGATTGCTCTAATTGGTGACCCAAAGTTGG

>GW_rep_c2047

TGCCTTGCCTCCCACGACTGCCCATCCATTATCAGCATGGAAGTATAGSTCGRACTGCTAGAAAGAACTCTCGGACTCACAAGGTAAATTACATGTACTCCAAAGCTGGACTAAGAATAGCATCTAAAGAACGTGACGACAGTAGCTTAGTTAGCTAGACATCATGATTTGGTCACACGCCTGAAGGGCCTGGTTTGCATAGTATCGTACATCCACATCAGGGTCCTCGCTGAGCTCGACGAGGCATGGCTTCACAGTCTTCTCAGCGACAGATTGATCAAGGATTGGTACAAGCGACTGCAGAACTTTCGCAACATTGAACTTGATGTTCGGCACTCTGTCCTTCGAGGAATTAATGACGACAGGGAGCAGCTTTTGGCATGTTATTTCTGCACCCATGACAGGGGCTAGCAATGAGATAGCTTGCAGGATGGTCATGCGATACAGATAATGTGGGTTGTTTATCTTCTCCAACACCTGAGGAATTATATGTTGCATTGTCCACTCTGGACCAAACTCCTCCGCCAGGCGCTTCAAGTTGTTTGCAGCAGCTTCTCTGATCGAGAAAACCTTATCTTCCAACCATTGCATGCAAAGTCGCCCCCAGCTTGTCATCAAAAAACCCAACACCTAACTGACTAGCCAGCAGAGGGATGTACTCAATTATTGCAAGGCGAACCCTCCAGTGCCTATCCTCCGCAAGTTCTACAATAGCCGGCAATAGAGATTGCGACAGCAAGTCAATTCCAATGACCTGGTTAACTTGATCAAGCTTGCTGATTATGTTGAGTCGAACATCAGGAAATTCATCCTTCAGCAAAGAAAGAAAAATAGGAAGAAGTTGTTCAATGGTAGCATCCTTCCCCAAGACAGGGGCCATTCCCATGATGACTGAAGCCAAAGCGGAGCGAACATGCTGAGACGAATCTGATGATAATTCCTTAACACACGGCAGAATGTGCTGAATTGCAAGCTGTGGACTTAATATCCTACAGAACTTAGTAACTTTCCGGCAGCTGCTATCCGCACTTCAGCCTCATTATCACGAAGGAGGCGAACATATGCAGGCACCAGGTCCGCTCTTGTAGGCTCGGGGCCAACTGCCTCGCAGAGCTCATACAATTGATTTGCAACCATATAACGGACACGCCAAGATTTATCCTGGGAGAAATTGACAA

>GW_rep_c11679

ACATAGTACAGCCATGGCCCCCACCGTGATGGCCTCGTCGGCCACCTCCGTCGCTCCTTTCCAGGGGCTCAAGTCCACCGCCAGCCTCCCCGTCAGCCGCCGCTCCAACGGCGCTAGCCTCGGCAGCGTCAGCAACGGTGGAAGGATCARGTGCATGCAGGTGTGGCCCATCGAGGGTATCAAGAAGTTCGAGACCCTGTCTTACTTGCCACCGCTCWSCACGGAGGCCCTCCTTAAGCAGGTGGACTACCTGATCCGCTCCAAGTGGGTGCCTTGTCTCGAGTTCAGCAAGGTTGGGTTCATCTTCCGTGAGCACAATGCATCTCCTGGGTACTACGATGGCCGATACTGGACAATGTGGAAACTACCTATGTTTGGGTGCACCGACGCCACACAGGTGCTAAAGGAGGTGGAGGAGGTCAAGAAGGAGTACCCTGACGCCTATGTCCGCATCATCGGTTTCGACAACAATCGTCAGGTGCAGTGCGTCAGCTTCATCGCCTTCAAGCCACCAGGCTGCGAGGAGTCCGGCAAGGCCTAAACAGCTCACTGACGACGACRGCCACATATAAAGTGCCATTGCAGTTTTGTCAACTCTGACATTGCTTTGGGTTTTCCTTCTCCATTTATCTTTCTTATTTGTTCCTAAGAATATGTGTATGTCCATGTTCATGTACCAACATGGCTCGAGAAAGCATGCTCGTATGTGAATGCTATCGG

>GW_rep_c37136

CTGCTGCTGATAGAGAGGAGAGGGAGATGGAAGGAGGAGGTGCGGAGGGCGTGACGAGGGTGCTGCTGGTGGACGACTCCCGGTCGACAGGAAGGTGGTGGAGCTGGTGCTCGGCAGCAACACCTTCGCCGGCTCCTTCCACGTTATCGCCGTCGACAGCGCCAAGAAGGCCATGGAGTTCCTGGGGCTCAAGGACGGCAAGGAGCAGGCCGTCGACATGGTGCTCACTGACTACTGCATGCCTGAGATGACCGGCTACGACCTTCTCAAGGCCATCAAGGCCATGAGTCCCCTCAAGCCGATCCCGGTGATCGTCATGTCGTCGGAGAACGAGCCCCAGAGGATCAGCAGATGCCTCAAGGCCGGCGCTGAAGATTACATCGTGAAGCCTCTTCAGAGTAAGGATGTGCCGCGCCTGAGGAGCTGCTCCAACGCCAAGCCGAAGGACCCCCCATGCAGCACTGTGAGCAAGAGTGCGGACCATATAGCTGCTGTCGATGGCACGTCGTCACTGCGACGGCGAGCACACCTCACCGATATCGCCATGGTCCTCCACTCGTCGAGCGCCGGGCTCTCGCACTACTTTCCGTTCCTCTTCAAGTTCATCCTGCTGGTCTACGCCATCCTGTGCGTGGGCGAGCTCCTGCACAGATGGTCAAACGGCTGCTTCCTCTCCTCCCTGTGGTGATGGTGCTGCAGTCCAGTGACATGCTGCACCTCTGCAAGGAGGAGCAGCTCTTGTGTTGTAGCTTATGAGTGAGCAGTGAACCTCCATGTTTCAACACCATGGATGGAGCTTTAGGTAGTAGATTCGTTTGCTTTGAGATTCTAACCGGGCGTCGGTTCTTGGCTTGTTGGGCTGACGAGTTGAGGAAGAACCCCTTGTATCCATCCTTGTACAAAACGCACAATGAGGCAGGCAGTATCTGATGTCCCTTTTGGCTCGGATCTTGGCTGATCTACTGATGTGACATTCAGGGAACTTTTCTTCGCCTTTCACTTGGTTGAGGGCGACTGCTCACCTGTTTCAATAGTTCAGATCTCATGAGAAAAGAACAGCACAAGTTGACTTAAAAGTAGCACCAGATCTTCTGATGGGAAAAGAAGAAGAAAATGCAGACACCCTAGTATGGTCTCAAAGGTCAGGT

>GW_rep_c6561

CCCGGGACGTTATCGCCACTACCAAGGGAGGGGATGGTCGGCCCTCTACTTAAAGTCCTCCTCGTCGTTAGTAACCTCCTACAAGGCGAGACTGTATTCGTCCACTTATTTTTTGTTTTGTTGTCCAACCTGCTTGCGGACTAAATTGTCGATACATGCTCATAGCAGTGATGCCAACAATTTTTATGAATGGTACATGCCATCCCCCATGATGAGAGCTATGCATATGAAGGACCTTGTAACCGTACAAACTTTTTCATGCTGCTTTCTGGTACTTTTTGCAGGGTACCAATCCCCCTGTTTTTACTGATGAGTGGCCACATTATTCCCCATGAAATAATTGCACCAAGGAGGGTAGAGAGATTTACTATATGAGGGCAAATCATCCCGGCACCAACGTATGTCATGCTAAAGTCAAAGTAGAATGTCTGCTTCCAGGCCTTCAAACCGAAAGTAGGGAACTGAACAAATCCACAAGCATCGCCTCCGGTGTAGAACCATTGGAAGAAACTCCATAGAAAGCTACCCCCAAAGAATTTCAGGAATCCACGGATTTGCTTCCTTGAATTCTTGTCCCCTTGATCGGTATGAAACCCGTTTATAAGAATAGCAGTTGCAGTCCCACTTGGGTAAACTAATTTGTAGTCGACGACCAATACCTGTCTCAGGGGAATCAAAGTGAGGAGCCCCCCGAAGCTGCAAGCGAGGAGGAATCCCGTCATCCAGCCTATCCCTGGCTCCTTCCAGCTTCCCGGCACGTTGCCCGGCGAGTCCCCGGCCAGCTCGTACGTCTTCTTGTTCAGGCCCAGCAAGGTTGACCCGAACCCACCGGCGAACGCGATGGTGTAGCAGGCCACGCCGCAGGTCTGGACGATGGTGTTCTCCTGCCGCGTGAAGGGGCGGGACACGATGCCGAAGCGATCCAGCAGGCGCGTCCACCCGCGGAGCGCCAGGAAGGAGAGCAGCGCGGCGGAGACGTTCAGCGTAGGCACCAGCCCGGTGGTGAGCGCGATCTTCATGACGATGACGGTGTAGATGAACCCGATCAGCAGCGCCGCCACCATGCCCCTCACCGTCAGCTCGTCCT

>GW_rep_c49894

AGTACACCTATGCTGAGTTGGAGAGGGAGCTGTACTGGCCATCCGAGAAGCTGAGAATCTCGGTTACTGGAGCTGGTGGTTTCATTGGATCCCATATTGCTCGCCGTCTGAAGAGTGAGGGGCACTAATCATTGCCTCGGACTGGAAGAAGAACGAGCATATGACTGAGGACATGTTCTGCCATGAGTTCCACCTTGCTGACCTCAGGGTCATGGACAACTGCCTTAAGGTACCAGCAACGTCGACCATGTCTTCAATCTTGCTGCTGATATGGGAGGCATGGGGTTCATTCAGTCCAACCACTCTGTTATCATGTACAACAACACCATGATCAGTTTCAACATGCTTGAGGCCGGGCGTATCAACGGTGTGAAGAGGTTCTTCTATGCCTCGAGGCATGCATCTACCCTGAATTCAAACAGCTTGAGACAAATGTGAGCTTGAAGGAAGCTGATGCCTGGCCTGCTGAGCCACAAGATGCCTATGGTTTGGAGAAGCTCGCGACCGAGGAGCTGTGCAAGCACTACACCAAGGACTTTGACATTGAGTGCCGTATTGGCCGTTTTCACAACATTTACGGTCCCTTTGGAACATGGAAAGGCGGTCGCGAGAAGGCACCAGCTGCCTTCTGTAGAAAGGCTCAGACCTCCACCGAACGGTTCGAGATGTGGGGTGATGGTCTCCAGACTCGATCGTTCACTTTTATCGACGAGTGCGTCGAGGGTGTTCTGAGATTGACAAAGTCGGACTTCTTGAGCCCRGAACWTTGGAAGTGATGAAATGGTGAGCATGAATGAGATGGCTGAGATTGTTCTCGGCTTTGAGGACAAGAAGCTGCCCATCCACCACATCCCTGGTCCAGAGGGTGTCCGCGGCCGCAACTCTGACAACACGCTCATTAAGGAGAAGCTTGGCTGGGCCCCCACAATGAGGCTCAAGGATGGCCTAAGGTTCACCTACTTCTGGATTAAGGAACAGATCGAGAAGGAGAGGACCGAGGGGATGGATGTCGCCCGGTACGGATCATCCAAGGTGGTGTCCACGCAGGCGCCGGTGCAGCTGGGCTCCCTCCGCGCGGCTGACGGGAAGGAGTAAACTATGCAGACCAACGCCAGGCCTATTACGCCAGTGCCCAAGTGCCCATCGATCGGATTCTTC

>GW_rep_c34820

CGTCCGCCACGATGGCGGCACCGGCGGCGGCGGCCGTGGGGAGGACGCACTACGAGGTGCTCGGGGTGGGCGCGGGAGCCAGCAGGGGGGAGATCAAGGCGGCCTACCGGCGTCTGGCCAGGGAGGTTCACCCGGACGCTGCTGACGGCGGTGGCGACGAGGGGTTCATCCGGCTGCACGCGGCCTACGCCACGCTGGCCGACCCCGACGAGCGCGCGCGCTACGACCGGGACGTGCCGGCCGCGCCGCGGGGATGATGATGGGCGGGCGGTGGCGACGTCCGGACCGGGCGTTCCGGCGGAGGACGTGGGAGACGGACCAGTGCTGGTAGGACGTACGAGCCGGGCAAACGCTGCTGCCCTCCGGGGGCTCCGAACGGCTGCCGTCCGGTTCGCTCAGGCACGGCGCGGCGTGGAGGTCGCGGCCGCTGGCGAAGGTCCTGCTGACTATCGTGCACCGTA

>GW_rep_c1723

CAGACGGGGATTTTGAATCTCCAAATGGGCTTTCGTACAATGACTGGACAGAATATGCTGTACCTGTCAACAGTATATCAGGTCTTGTTTCTTCACCAGGCACTTATTGCTGCAGAGTACTGTGGGGTCAAGGTTGAGCTGACCAAGAACTTTGAGATGGGTGTCTCAAACAAAACCCCTGAATTCGTCAAGATGAATCCCCTTGGGAAGTTCCTGTTCTTGAGACTCCTGATGGTGCTGTTTTTGAGAGCAATGCTATTGCACGCTATGTTGCTCGCTCAAAGGGTGACAACTGCTTTGGGTGGTTCTCTTATTGAATATGCTCGTGTTGAGCAATGGATGGACTTCGCTGCCACAGAGGTTGATCCCAATATCGCAAGGTGGTTGTACCCAAGGCTTGGTTATATGATTTACAATGCCCAGTCTGAGGAATTTGGCATTACTGGATTAAAGAAGGCCCTTGATGCATTGAACACACACCTCGCCTCAAACACATTCCTTGTTGGGCATTCTGTCACTCTGGCTGATATTGTCATGACATGCAACCTCTACCATGGTTTTGCGCGGATCTTGACCAAGACTTTCACATCTGAGTTCCCTCATGTTGAGAGGTACTTCTTAGTCGTGGGAGGCAAGGCA

>GW_rep_c33890

CCGCGTCCCCCTCGTCCGCCGCCACGCCCCGCGCCGCCTCCTTCGCCTGCGCGGTCGCGCGCGGGCTCCCCTCCCTCCGCCTCGCCGCGCCCCGCCGCCGCCGCGGCGACCTCGGCAGGCCCAGGGCCGCCGGCGCCGAAGCCGCGGCCGAGAACTACGCGACCGCGCTGACGGAGGTGGCCGCCGAGAGCGACTCCCTCGACGCGACTGTCGCGGACATGGAGAAGCTGGAGAAGATCTTCGCCGAGGAGGCCATCGCCGAGTTCTTCGACAACCCCACCGTGCCGCGCGAGGAGAAGACGGCGCTCATCGACGAGATCGCCAAGTCGTCGGAGCTGCAGCCGCACACCGTCAACTTCCTCAACGTGGTCATCGACAACACCCGCGCGGGCCTGATGCCGCAGATCGTGCGCGAGTTCGAGACCGCCTACAACGCGCTCACCGGCACCGAGGAGGCCGTCGTCACCTCCGTGGTGCAGCTCGAGTCGCAGGACCTCGCTCAGATCGCCACGCACGTGCAGAACATCACCGGCGCCGCCAACGTCCGGATCAAGACCCGCCTTGACCCGGAGCTCATCGCCGGCTTCACCGTGCAGTACGGCCGCGACAGCTCCAACTTCATCGACATGAGCGTCCGCAAGCAGATCGCGGAGATCACCTCCGAGTTCGAGATGCCCTCCATTAACCTCGAAGTCTGATGCTTAGGTCCTCTTTTTTCTGCCGTCTCTATACATGTTCTTCTGTATCATTATCCGATGAAATGTTAAATACTCCCTCCGTCCGGAATTATTTGTCGCATAAATGGATAAAAATGGATGTATCTAGAACTAAAATACRTCTAGATACATCCATTTCTCCGACAAGTATTTCCGGACGGAGGGAGTAAATGAAAGCAGTGGTGTGTGAGGAGGGAGACCAGAATTCTCTGGCAAAACAATGTCATGGTTGGGAGAATCATTCAAAAACATTGTCAGTTCGTCCAATAATATTCCATCAATTTCAGGAGGCGCATTATTGTCCTCATTAGAACAGATATTAACTTGTTCAGAAAATTGAACAGACTGCTGAGGAGCGGCTGGTTCCTGAACAACTATGGCAGTAGCCAAGCCAGAACTTTCGATTTGTGGATCCTCTACACCTGACGAGGGTGCAAAAGAGAAGATAGAACTTCCAACATCCAAATTTTCCCATTCATTTTCATCGAAAGGAGCCCCATATTGCTCACCAATCCTTGGGCCAAGCCCACTCTTTTTAAATATCTTGCAGAGTACATAAGCATCCTTAGAGAAACCGCAGACACCAAAGTTTCATCTTCCATTTTGATTCATACATTACCCA
