# Supplementary figures and images for: Transcriptome Exploration in Leymus chinensis under Saline-Alkaline Treatment Using 454 Pyrosequencing
Source: PLoS One. 2013 Jan 24;8(1):e53632. doi: 10.1371/journal.pone.0053632 (PMC3554714; doi:10.1371/journal.pone.0053632)

**Text S2 The original RT figures of 16 validated genes.**


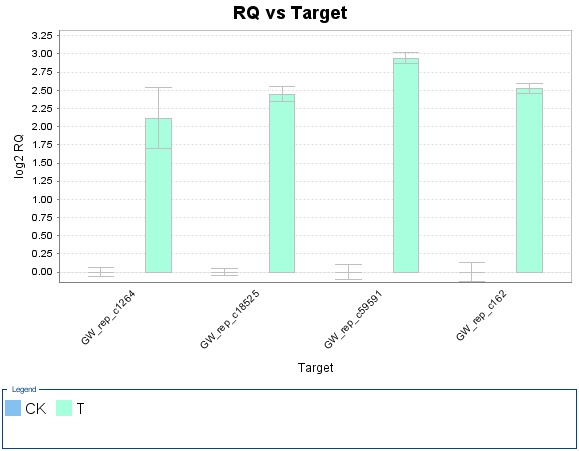

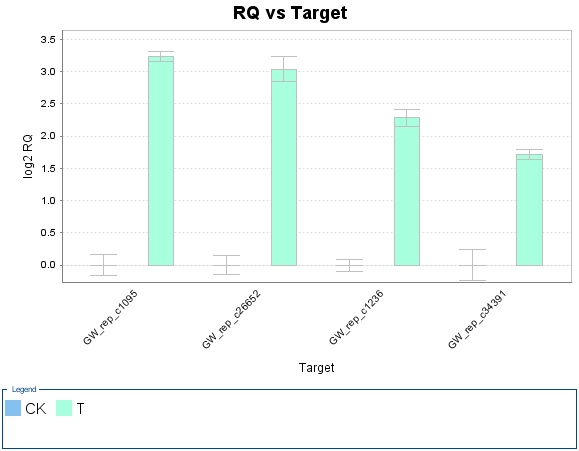


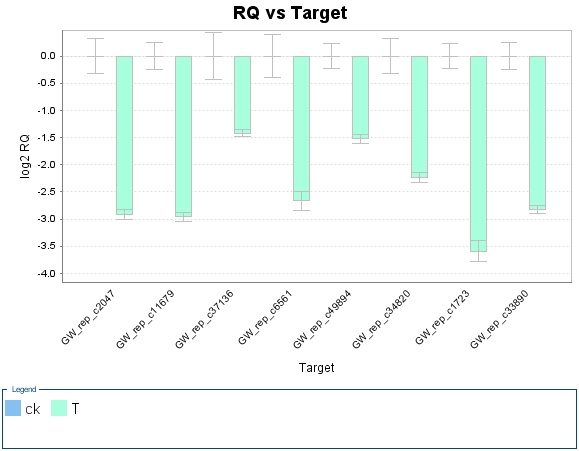

Supplement: Text S3 — The original RT figures of 16 validated genes. (DOC) [file pone.0053632.s007.doc]
